# 810 Development of a Porcine Burn Wound Model

**DOI:** 10.1093/jbcr/irae036.350

**Published:** 2024-04-17

**Authors:** Adam J Singer, Steve McClain

**Affiliations:** Stony Brook University, Smithtown, NY; Stony Brook University, Smithtown, NY

## Abstract

**Introduction:**

Deep partial thickness burns generally require more than 2-3 weeks to heal often resulting in hypertrophic scarring and patient morbidity. Because the skin of pigs most closely resembles that of humans, the pig is often used as an experimental burn wound model. We describe the development of a reproducible burn wound model in Yorkshire pigs.

**Methods:**

We created standardized 5 x 5 cm burns on the backs and flanks of anesthetized domestic pigs (30 kg) using a radiant heating device set at 600 degrees C and applied to the animal’s skin for 5, 10, or 15 seconds. Full thickness 4 mm punch biopsies were obtained from the burns at 4 and 24 hours for burn depth evaluation. The tissue sections were stained with Hematoxylin and Eosin, HMGB-1 (a marker of necrosis) and activated cleaved caspase 3 (a marker of apoptosis). The tissue sections were viewed by a board-certified dermatopathologist blinded to duration of exposure. The primary outcome was the deepest level of injury to the blood vessels or epithelial appendices (hair follicles or apocrine glands).

**Results:**

Exposure of the pigs’ skin to the heating device for periods of 5, 10, and 15 seconds resulted in superficial partial, deep partial and full thickness burns respectively. Average depth of dermal injury 24 hours after exposure was 0.4 mm, 1.3 mm, and 2.4 mm for exposure times of 5, 10 and 15 seconds respectively.

**Conclusions:**

We describe a standardized porcine model for creating reproducible superficial partial, deep partial and full thickness burns.

**Applicability of Research to Practice:**

This model is now being used to investigate novel therapies for deep burns.

